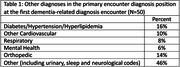# Mild Cognitive Impairment and Dementia Diagnoses after an Annual Wellness Visit in a Large Primary Care Network

**DOI:** 10.1002/alz.089864

**Published:** 2025-01-09

**Authors:** Monica Zigman Suchsland, Annette L. Fitzpatrick, Jaqueline G. Raetz, Sarah McKiddy, Amy P Hsu, Barak Gaster

**Affiliations:** ^1^ University of Washington, Seattle, WA USA

## Abstract

**Background:**

The increasing incidence of cognitive impairment in older adults poses significant, complex challenges for patients and their families. While specialists have traditionally addressed this care, there is an urgent need to shift diagnosis and management of mild cognitive impairment (MCI) and dementia to primary care. The annual wellness visit for Medicare recipients provides an excellent opportunity for primary care providers (PCPs) to assess patients who may need a cognitive evaluation.

**Method:**

We aimed to describe the timing of dementia‐related diagnoses in a large primary care network from electronic health record (EHR) data. All coded encounter data for patients 65 years and older seen in one of 14 primary care clinics at the University of Washington from January 1, 2019‐ August 1, 2023 were extracted from the EHR system and summarized.

**Result:**

A total of 40,524 medical encounters for a Medicare annual wellness visit (AWV), billing code of G0439, were identified representing 17,179 unique patients. Of these, 187 (1.1%) had a first diagnosis of MCI, dementia or other cognitive impairment based on ICD 10 codes at a following clinic visit. The top diagnoses included dementia unspecified (36.9%), impaired cognition or other pre‐dementia diagnoses (26.2%), and Alzheimer’s Disease (23.0%). A median of 287 days (IQR 0‐587 days) elapsed between the AWV and coding of a dementia‐related diagnosis. Among encounters during which new dementia‐related diagnoses were documented, in 73% the primary diagnosis of the visit was a dementia‐related ICD‐10 diagnosis code; 27% listed other primary diagnoses (Table 1). Following a new dementia‐related diagnosis, a median of 3.3 (IQR 2‐5) primary care visits/year were made. Major comorbidities recorded in these visits included hypertension, diabetes, other cardiovascular conditions, chronic pulmonary disease, renal failure, other neurological disorders, and depression. The median weighted Charlson Comorbidity Index was 2.0 (IQR 0‐2).

**Conclusion:**

These data may be helpful baseline measures to evaluate future trends in dementia‐related diagnosis following AWV. As programs designed to provide training and support for diagnosis and management of cognitive impairment in primary care settings are implemented, it will be important to understand the role the AWV serves as a tool for PCPs.